# Detection Of Four Dengue Serotypes Suggests Rise In Hyperendemicity In Urban Centers Of Brazil

**DOI:** 10.1371/journal.pntd.0002620

**Published:** 2014-02-27

**Authors:** Christian Julián Villabona-Arenas, Jessica Luana de Oliveira, Carla de Sousa Capra, Karime Balarini, Mauricio Loureiro, Celso Ricardo Theoto P. Fonseca, Saulo Duarte Passos, Paolo Marinho de Andrade Zanotto

**Affiliations:** 1 Laboratório de Evolução Molecular e Bioinformática, Departamento de Microbiologia, Instituto de Ciências Biomédicas, Universidade de São Paulo, São Paulo, São Paulo, Brazil; 2 Laboratório de Saúde Pública, Secretaria da Saúde, Prefeitura Municipal de Guarujá, Guarujá, São Paulo, Brazil; 3 Itapema Laboratório de Análises Clínicas, Guarujá, São Paulo, Brazil; 4 Laboratório de Infectologia Pedriátrica, Faculdade de Medicina de Jundiaí, Jundiaí, São Paulo, Brazil; Baylor College of Medicine, United States of America

Dengue fever is the most common infectious disease transmitted by a mosquito and a major economic and disease burden in endemic countries. The reported number of dengue cases in 2013 evidences the disease's disturbing impact on human health in Brazil: 950,193 clinical cases, 3,749 with severe manifestations, and 201 deaths (data up to July 13, 2013 by the Pan American Health Organization); any of the four serotypes was the etiologic agent in different geographical regions. The epidemiological surveillance center “Prof. Alexandre vranjac” documented in the state of São Paulo a total of 105,973 cases (data up to May 6, 2013). This state is the major industrial and economic powerhouse of Brazil, and its capital is the largest city in the southern hemisphere. In this state, the municipality of São Paulo has 11,253,503 inhabitants and 7,388 inhabitants/km^2^ and is the country's main international center of business, culture, and tourism. Because overcrowded cities present highly favorable conditions for increased transmission, we are deeply concerned that hyperendemicity (the cocirculation of multiple dengue serotypes) may become established in this largest interconnected urban area in Brazil.

Hyperendemicity was first associated with increased transmission and the emergence of severe dengue in Asia in the 1950s and then in the Caribbean in the 1980s [1]. Nevertheless, a similar epidemiological pattern of cocirculation in the most highly populated urban areas of Brazil has not been reported so far and is of utmost relevance, since distinct genotypes from the four serotypes have been reported in Brazil. Just to recapitulate, in 1986 DENV-1 was introduced in the country, causing outbreaks. The first autochthonous cases of DENV-2 and DENV-3 were detected respectively in 1990 and 2000 [2]. DENV-4 was isolated for the first time in 1982 in a focal epidemic in the northwestern region of the Brazilian Amazon. Later, in 2008, this serotype emerged as an important pathogen during the Brazilian outbreaks from 2010 to 2011 [3]. Remarkably, in 2011, Bastos et al. [4] detected the simultaneous circulation of all four dengue serotypes in the municipality of Manaus, which has 1,802,014 inhabitants and 158 inhabitants/km^2^ and is located in the middle of the Amazon rain forest, in the northern state of Amazonas.

We expect a change in the epidemiological pattern once endemic hyperendemicity and high infection rates lead to an immune population before adulthood. Hence, children less than 16 years old will be at greater risk for dengue [5]. For example, prior to 2007, the disease affected mostly adults (20- to 40- year-old people). Nevertheless, during the 2007–2008 epidemics, over 53% of the cases affected children under 15 years of age [6]. Consistently, Rodriguez-Barraquer et al. [7] argued, and we agree, that the disease's shift towards younger patients observed in Brazil can be partially explained by the accumulation of immunity against multiple serotypes in older people. Therefore, we now have a situation where dengue infections in children have the potential to become a leading cause of hospitalization and death.

Additionally, we need to take into account that simultaneous or sequential epidemics with different serotypes are a common risk factor associated with severe cases [1]. Severity is possibly due to antibody-dependent enhancement, even though the risk is reduced after infection with two or more serotypes [8]. Severe dengue illness has been seen mainly in infants in Asia, where the presence of circulating dengue antibodies acquired passively by maternal vertical transmission is a frequently reported risk factor [9]. Nonetheless, in Brazil, the sequential introduction of serotypes has been accompanied by mild cases rather than severe ones. In this respect, Halstead [8] argued that this may reflect a failure of clinicians to perform diagnoses that fulfill the requirements of the WHO case definition or be due to local human genetic resistance.

To corroborate if hyperendemicity is being established in a populated area in the country, we determined whether one serotype or multiple ones caused the 2013 epidemic in some critical localities in the state of São Paulo. In collaboration with our public health authorities, we collected acute-phase sera from suspected dengue patients from the cities of Guarujá (located in the coastal region with 290,752 inhabitants and 2,035 inhabitants/km^2^) and Jundiaí (located in the mountain range with 370,126 inhabitants and 856 inhabitants/km^2^) from December 20, 2012 to May 2013 (summer months). Jundiaí in the west and Guarujá in the east (seaside) are adjacent to, and tightly interconnected with, the densely populated municipality of São Paulo. We expected that this approach would inform on the cocirculation of viruses in the entire metropolitan area. Viral RNA was extracted from sera of 24 positive samples selected at random (20 from Guarujá and the only four samples that tested positive from Jundiai), and we amplified and sequenced the capsid/premembrane junction that was proposed by Lanciotti et al. [10] for typing DENV. All sequences determined in this study were deposited in GenBank (427 bp; accession KF286626-KF286649). To help in classifying our sequences, a small time-stamped dataset comprising 35 sequences that were representative of both serotypes and genotypes was retrieved from GenBank and aligned with our sequences. A phylogenetic tree (Figure 1) was built using a Bayesian approach, and our evolutionary estimates matched those of Twiddy et al. [11], which validated our analysis. Sequences from both cities belonged to different serotypes. Remarkably, samples from Guarujá clustered within the four serotypes, while samples from Jundiaí grouped with either DENV-1 or DENV-4. We will argue that these findings corroborate a change in epidemiological pattern accompanying a rise in Brazilian urban hyperendemicity that constitutes a greater challenge for surveillance and control. Crucially, the presence of two serotypes in the same outbreak may be considered as an important warning for high levels of transmission, since Jundiaí has no significant historic record of epidemics.

**Figure 1:**
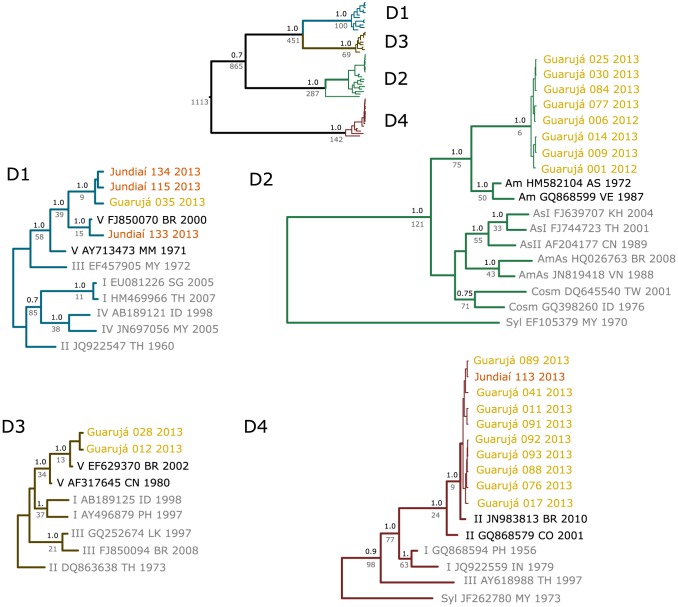
Maximum credibility tree (and its four serotype subtrees) showing the evolutionary relationships among the capsid/premembrane junction sequences of 55 strains. Posterior probability support values are shown over the nodes and TMRCAs values below the nodes. Reference sequences abbreviated by serotype (D1–4), genotype (*Am*: American, *AmAs*: American/Asian, *As*: Asian, *Cosm*: Cosmopolitan, *Syl*: Sylvatic, *I*, *II*, *III*, *IV*, or *V*), GenBank accession, ISO code for country, and isolation year.

The presence of the four serotypes in a single outbreak in one of the most densely populated areas of Brazil is a disturbing finding that has also been documented in Asian countries, particularly in India, which has the largest dengue burden in the world. Recurring dengue epidemics in that country resulted in the establishment of hyperendemic areas, typically in large, densely populated cities, where most DENV serotypes circulate in a sustained fashion [15]. Roughly two years after the report on the presence of DENV-4 in Manaus (Amazon), we now find the four serotypes cocirculating in the south of the country in the outskirts of the municipality of São Paulo. Therefore, a continued advocacy of long-term prevention and control is imperative. Our concern is that if we ignore the urban hyperendemicity, children will be at greater risk for severe disease [5].

## Ethics Statement

Both the Human Research Ethics Committee from the Biomedical Sciences Institute of University of São Paulo and the Research Ethics Committee from the Faculty of Medicine of Jundiaí approved the study; a written informed consent was obtained from all patients.
